# Kidney Injury in Adults After Hematopoietic Stem Cell Transplantation: Acute Kidney Injury, Transplant-Related Complications, and Long-Term Outcomes

**DOI:** 10.3390/cancers18213510

**Published:** 2026-10-31

**Authors:** Lamis Almalki, Nina Weber, Anup Devasia, Abhijat Kitchlu

**Affiliations:** 1 Department of Nephrology, Prince Sultan Military Medical City, Riyadh 11159, Saudi Arabia; 2 Division of Nephrology, Stadtspital Zürich, Tièchestrasse 99, 8037 Zürich, Switzerland; 3 Division of Hematology, The Ottawa Hospital, Department of Medicine, University of Ottawa, Ottawa, ON K1H 8L6, Canada; 4 Division of Nephrology, University Health Network, Department of Medicine, University of Toronto, Toronto, ON M5G 2C4, Canada

**Keywords:** hematopoietic stem cell transplantation, acute kidney injury, chronic kidney disease, transplant-associated thrombotic microangiopathy, sinusoidal obstruction syndrome, graft-versus-host disease, calcineurin inhibitors, nephrotoxicity, onconephrology

## Abstract

Kidney complications are common after hematopoietic stem cell transplantation (HSCT) and contribute substantially to short- and long-term morbidity and mortality. Acute kidney injury (AKI) affects a large proportion of recipients, particularly after allogeneic transplantation, and reflects the combined effects of baseline patient vulnerability, conditioning intensity, calcineurin inhibitor exposure, infection, hemodynamic disturbances, and transplant-specific complications. This review summarizes the epidemiology, risk factors, and major causes of kidney injury after HSCT, with emphasis on a time-based diagnostic approach. Important etiologies include sepsis, marrow infusion syndrome, sinusoidal obstruction syndrome, graft-versus-host disease-associated kidney injury, transplant-associated thrombotic microangiopathy, and glomerular disease. We also discuss emerging therapeutic approaches, including complement-directed therapy for transplant-associated thrombotic microangiopathy and defibrotide for sinusoidal obstruction syndrome. Beyond the acute transplant period, AKI is increasingly recognized as a major determinant of subsequent chronic kidney disease, cardiovascular risk, and impaired long-term survival. Structured surveillance of kidney function, blood pressure, and proteinuria, together with early recognition of transplant-specific syndromes and closer nephrology follow-up in high-risk patients, is therefore essential. Multidisciplinary collaboration between transplant hematology and nephrology is central to improving kidney and overall outcomes across the HSCT continuum.

## Simple Summary

Kidney complications are common in adults who receive a stem cell transplant, which is a treatment for blood cancer and other serious blood disorders. About half of patients develop sudden kidney injury within the first three months, and some go on to develop lasting kidney disease, which is linked to shorter survival. The causes include infections, medications, and complications of the transplant itself, such as blocked small veins in the liver, damage to small blood vessels, and immune reactions. This review explains how the timing of kidney injury after transplantation points to its likely cause, how to measure kidney function reliably in these patients, which treatments are available or still being tested, and how survivors should be monitored with regular checks of kidney function, blood pressure, and protein in the urine. Teamwork between transplant doctors and kidney specialists may help protect kidney health and improve survival.

## 1. Introduction

Worldwide, more than 90,000 hematopoietic stem cell transplantation (HSCT) procedures are performed annually, and approximately 1.5 million transplants were completed between 1957 and 2019. Although the use of allogeneic HSCT has increased more rapidly recently, autologous transplantation remains the predominant procedure. The most common indications for allogeneic HSCT are lymphoproliferative disease and leukemia, whereas multiple myeloma is the predominant indication for autologous HSCT [1,2].

Advances in transplantation strategies and supportive care have substantially improved overall outcomes after HSCT. In contrast, the burden of kidney disease has changed little over the past two decades. Acute kidney injury (AKI) and chronic kidney disease (CKD) remain among the most common non-relapse complications after transplantation and are associated with significantly increased short- and long-term mortality, particularly in patients with severe AKI [3,4]. Renal dysfunction after HSCT may be multifactorial, reflecting the potential effects of nephrotoxic medications, infectious complications, engraftment syndrome, hepatic sinusoidal obstruction syndrome, thrombotic microangiopathy and graft-versus-host disease (GVHD). The risk of AKI is further influenced by both patient- and transplant-related factors, including underlying disease, age, comorbidity burden, performance status, transplant type, conditioning intensity, stem cell source, degree of human leukocyte antigen (HLA) mismatch, and the immunosuppressive regimens used for GVHD prophylaxis [5,6,7,8].

The clinical importance of AKI following HSCT is underscored by its high incidence and profound impact on survival. In a meta-analysis of 36 cohort studies published between 1995 and 2019, encompassing 5144 adult patients, Kanduri et al. reported a pooled AKI incidence of 55.1% (95% confidence interval (CI) 46.6–63.3%) across all AKI definitions, with stage 3 AKI occurring in 8.3% of patients. AKI was associated with markedly increased mortality, with pooled odds ratios of 3.05 (95% CI 2.07–4.49) for 3-month mortality and 2.23 (95% CI 1.06–4.73) for 3-year mortality [3]. More recent studies have reported comparable AKI incidences ranging from approximately 33% to 64%, with most episodes occurring early after transplantation (median onset day 12–13) and cumulative incidences generally assessed within the first 100 days [6,7,9,10].

Reported AKI incidence varies considerably across studies, in part because of differences in diagnostic criteria. The subsequent development of the Risk, Injury, Failure, Loss of kidney function, and End-stage kidney disease (RIFLE), Acute Kidney Injury Network (AKIN), and ultimately the Kidney Disease: Improving Global Outcomes (KDIGO) criteria has provided a standardized framework for AKI diagnosis and severity classification, with KDIGO now representing the preferred definition in contemporary HSCT studies [3]. Together with variations in patient populations, transplant characteristics, and study design, these differences in diagnostic criteria likely contribute to the heterogeneity in reported AKI incidence [11]. 

This narrative review is based on a PubMed/MEDLINE search for articles on kidney complications after HSCT published up to October 2026, supplemented by screening the reference lists of relevant articles. Guidelines, consensus statements, randomized trials and prospective cohort studies were given priority, and the design of key studies is stated in the text.

## 2. Epidemiology and Risk Factors for AKI After HSCT

### 2.1. Assessment of Kidney Function After HSCT

Serum creatinine remains the basis of current AKI definitions but has important limitations for kidney function assessment in patients with cancer. Reduced muscle mass and malnutrition may result in low serum creatinine concentrations and consequently overestimation of kidney function. Cystatin C may provide complementary information, although determinants other than glomerular filtration rate (GFR), including inflammation, malignancy, and corticosteroid exposure, may affect its concentration. The recent Acute Disease Quality Initiative (ADQI) consensus therefore recommends combined creatinine–cystatin C-based estimated GFR (eGFR; CKD-EPI_cr-cys_) when feasible [12]. Accordingly, reported AKI incidence and associations with individual risk factors should be interpreted in the context of the kidney function marker and AKI definition used.

These estimating equations, including the combined creatinine–cystatin C equations, assume steady-state kidney function and should therefore be used to describe baseline or stable kidney function. During AKI, serum creatinine lags behind the change in GFR, so eGFR overestimates kidney function while it is falling and underestimates it during recovery [13].

### 2.2. Incidence According to Transplant Type

Transplant type is one of the strongest determinants of AKI risk after HSCT. The lower incidence observed after autologous transplantation is largely attributable to the absence of GVHD as well as the lack of exposure to calcineurin inhibitors (CNIs) and methotrexate used for GVHD prophylaxis [14]. Earlier studies demonstrated a substantially higher incidence of AKI following allogeneic transplantation than after autologous transplantation. In a landmark study by Ando et al., AKI occurred in 48% and 66% of patients receiving non-myeloablative (NMA) and myeloablative conditioning (MAC), respectively, compared with only 19% of autologous transplant recipients. This disparity was mirrored by mortality, which ranged from 50% to 54% after allogeneic transplantation compared with approximately 20% following autologous transplantation [11]. 

Similar differences have been reported in more recent cohorts. Munch et al. observed a 100-day AKI incidence of 20.7% among patients with multiple myeloma undergoing autologous HSCT and 35.2% among patients with Hodgkin lymphoma, whereas patients with leukemia receiving allogeneic transplantation experienced an AKI incidence of 81.8% [15]. These findings emphasize that transplant modality and the underlying disease are major determinants of post-transplant renal risk (see Table 1).

### 2.3. Conditioning Intensity and Regimen-Related Risks

Conditioning chemotherapy administered before donor stem cell infusion is one component of AKI risk after HSCT. Conditioning regimens are broadly classified as MAC, reduced-intensity conditioning (RIC), or NMA. In addition to inducing sufficient immunosuppression to permit donor stem cell engraftment, MAC regimens eradicate malignant cells and may augment antitumor immunity by promoting T-cell responses [16]. By contrast, RIC and NMA regimens rely more heavily on graft-versus-tumor effects and are therefore preferentially used in older patients or those with substantial comorbidity who may not tolerate intensive conditioning. Regardless of conditioning intensity, allogeneic HSCT recipients require post-transplant immunosuppression to prevent GVHD, whereas autologous recipients do not [14]. 

The relationship between conditioning intensity and AKI is complex, and current evidence does not support a uniform association between greater conditioning intensity and AKI risk. Conditioning intensity is closely linked to patient selection and transplant strategy, including baseline kidney function, age and comorbidity, underlying malignancy, donor characteristics, T-cell depletion, and choice of GVHD prophylaxis. Post-transplant CNI exposure, GVHD, and infectious complications further modify AKI risk. Consequently, comparisons between MAC, RIC and NMA are particularly susceptible to confounding by indication.

Individual cohorts have nevertheless demonstrated differences according to conditioning intensity. In a study by Parikh et al. comparing 140 patients receiving MAC with 129 receiving NMA conditioning, MAC was associated with an approximately fourfold greater requirement for kidney replacement therapy, despite older age and greater comorbidity in the NMA group [17]. A Spanish single-center cohort reported a higher 1-year cumulative incidence of AKI with MAC than with RIC (64% vs. 52%, *p* = 0.005) [8]. However, these findings should not be interpreted as establishing MAC as intrinsically more nephrotoxic. Abramson et al. reported the opposite association, with a higher incidence of AKI among patients receiving RIC than MAC. This was attributed, at least in part, to differences in transplant protocols, including the use of T-cell-depleted grafts and CNI-free GVHD prophylaxis in the MAC cohort, as well as a preferential selection of patients with greater comorbidity for RIC [7].

This trade-off between conditioning intensity, disease control and treatment-related toxicity is further illustrated by Adhikari et al., who analyzed 13,187 adult transplant recipients using a competing-risk framework. Compared with MAC, RIC was associated with a lower risk of major organ failure (MOF; adjusted hazard ratio (aHR) 0.72, 95% CI 0.64–0.82) but a higher risk of relapse (aHR 1.14, 95% CI 1.07–1.23), while NMA conditioning showed an even greater reduction in MOF (aHR 0.55, 95% CI 0.46–0.66) accompanied by a higher relapse risk (aHR 1.42, 95% CI 1.30–1.54). Importantly, MOF was a composite endpoint including dialysis-requiring renal failure, thrombotic microangiopathy, veno-occlusive disease (VOD), and idiopathic pneumonia syndrome; thus, these findings cannot be interpreted as demonstrating an independent effect of conditioning intensity on AKI specifically, but rather illustrate how conditioning intensity shifts the balance between treatment-related organ toxicity and disease control. Taken together, these findings suggest that conditioning intensity should not be considered an independent or uniformly directional determinant of AKI risk; rather, AKI risk reflects an interplay between regimen intensity, patient selection, transplant characteristics, GVHD prophylaxis, and post-transplant complications [18]. 

Beyond conditioning intensity, the specific agents comprising the conditioning regimen may influence renal risk. In a cohort of 987 patients undergoing RIC allogeneic HSCT, Vergara-Cadavid et al. found that fludarabine–melphalan conditioning was associated with a higher risk of AKI compared with fludarabine–busulfan or fludarabine–cyclophosphamide plus total body irradiation (TBI) (hazard ratio (HR) 1.35, 95% CI 1.01–1.81; *p* = 0.05) [6]. For high-dose melphalan (200 mg/m^2^), evidence to guide dose adjustments in patients with impaired kidney function remains limited, with no randomized controlled trials establishing an optimal dosing strategy. Clinical practice varies considerably between centers, and dose reduction to 140 mg/m^2^ is frequently considered in patients with reduced kidney function. In patients receiving hemodialysis, melphalan is administered after dialysis [19,20]. Fludarabine dosing is based on body surface area (BSA) using total body weight. As fludarabine is predominantly renally cleared, dose modification (dose reduction of 20% for creatinine clearance 50–79 mL/min and 40% for 30–49 mL/min; not recommended below 30 mL/min) is recommended to avoid hematologic and neurotoxicity [21]. Langenhorst et al. further demonstrated that fludarabine exposure is strongly influenced by kidney function and body weight and proposed individualized, exposure-targeted dosing based on eGFR and body weight or therapeutic drug monitoring (TDM) to reduce overexposure [22].

The association between TBI and AKI remains less clear; a Spanish single-center study and a pediatric cohort of 92 patients reported an increased risk of severe AKI associated with TBI, with HRs approaching 1.8 [8,23]. In contrast, in a prospective cohort of 147 HSCT recipients, Hingorani et al. found no association between TBI dose and the risk of acute renal failure [24].

### 2.4. Calcineurin Inhibitor Exposure and GVHD Prophylaxis Strategies

Beyond conditioning-related toxicity, exposure to CNIs is one of the most consistently identified modifiable risk factors for AKI after allogeneic HSCT. Several studies have demonstrated that supratherapeutic CNI concentrations are independently associated with increased risk of AKI, highlighting the importance of careful TDM and dose adjustment [6,7,9]. In addition, the combination of methotrexate with a CNI as GVHD prophylaxis has been associated with a 2.1-fold higher incidence of severe AKI [8]. Post-transplant cyclophosphamide (PTCy), initially established as GVHD prophylaxis in haploidentical transplantation, is now widely used across donor types. Its renal impact remains incompletely defined and likely depends on both PTCy exposure and the accompanying immunosuppressive regimen. In a recent randomized phase II trial, CNI-free prophylaxis with PTCy plus ruxolitinib was associated with substantially less grade 2–4 AKI than PTCy plus tacrolimus and mycophenolate mofetil (1.6% vs. 12.1%, *p* = 0.02), suggesting that avoidance of CNI exposure may reduce renal toxicity [25]. Conversely, PTCy itself may contribute to organ toxicity at conventional doses; reduced-dose PTCy strategies (40 mg/kg on days +3/+4) have shown low rates of renal and other organ toxicities in retrospective cohorts, although comparative data demonstrating a reduction in AKI are lacking [26].

### 2.5. Baseline Patient Characteristics and Risk Prediction

Patient-related factors substantially influence susceptibility to AKI after HSCT and contribute to both early renal complications and subsequent CKD [27]. Baseline renal function is an independent predictor of overall survival, non-renal mortality, and the subsequent need for kidney replacement therapy following allogeneic HSCT [6,8,28,29]. Increasing age is another well-established risk factor. In one cohort of 422 allogeneic transplant recipients, age > 55 years was associated with a 2.4-fold higher risk of severe AKI [8]. Similarly, Yanagisawa et al. reported that age ≥ 60 years independently predicted fatal renal complications (HR 1.465, 95% CI 1.241–1.730; *p* < 0.001) [27].

The influence of sex has been less consistent. Most studies identify male sex as a risk factor for renal complications [27,29], whereas Sun et al. identified elderly female sex as an independent risk factor for severe AKI among patients with acute leukemia [30]. Higher baseline serum albumin concentrations (>3.4–3.5 g/dL), likely reflecting better nutritional status, lower inflammation, and preserved oncotic pressure, have consistently been associated with lower risk of AKI after HSCT [6,7,9]. 

Pre-transplant comorbidity is an important determinant of post-transplant outcomes and is routinely incorporated into risk prediction models. The Hematopoietic Cell Transplantation-Comorbidity Index (HCT-CI) was specifically developed to improve risk stratification in transplant recipients and demonstrates greater sensitivity than the Charlson Comorbidity Index (CCI), identifying comorbidity in 62% versus 12% of patients while providing superior discrimination for survival outcomes. Within the HCT-CI, moderate/severe renal dysfunction (serum creatinine > 2 mg/dL, dialysis dependence, or prior kidney transplantation) is assigned 2 points (comorbidity weights range from 1 to 3), with total scores of 0, 1–2, and ≥3 defining low-, intermediate-, and high-risk groups, respectively [31].

An HCT-CI score of ≥4 was independently associated with an increased risk of AKI after HSCT (HR 1.43, 95% CI 1.14–1.79; *p* = 0.002) [6]. Although the HCT-CI was originally developed to predict overall and non-relapse mortality rather than kidney-specific complications, several studies support its value for identifying patients at increased renal risk [6,27]. In a nationwide Japanese cohort of 36,596 HSCT recipients, including approximately 16% pediatric patients, Yanagisawa et al. reported a 5-year cumulative incidence of fatal renal complications of approximately 2.2%, with most events occurring relatively early after transplantation. Moderate or severe renal comorbidity according to the HCT-CI, together with increasing age and advanced disease status, independently predicted fatal renal complications [27]. 

Several HSCT-specific prediction models for early AKI have subsequently been developed. Rodrigues et al. proposed an adult risk score incorporating four readily available variables: HCT-CI, pre-existing CKD, underlying disease (lymphoma or leukemia versus multiple myeloma) and the platelet-to-lymphocyte ratio as a marker of systemic inflammation [4]. Gan et al. developed a nomogram including donor source, HLA compatibility, ABO matching, tacrolimus exposure, acute GVHD, infection, and triazole antifungal therapy, achieving a concordance index of 0.842 (0.809 after internal validation) [32]. Although these models are promising, most remain limited by retrospective single-center designs and require external validation before widespread clinical implementation. 

## 3. Etiologies of AKI After HSCT

The causes of AKI following HSCT are diverse and vary throughout the transplant course. As the patient’s clinical condition and transplant-related complications evolve over time, the differential diagnosis changes accordingly. Therefore, a time-based approach provides a practical framework for understanding the major etiologies of AKI after HSCT.

**Figure 1:**
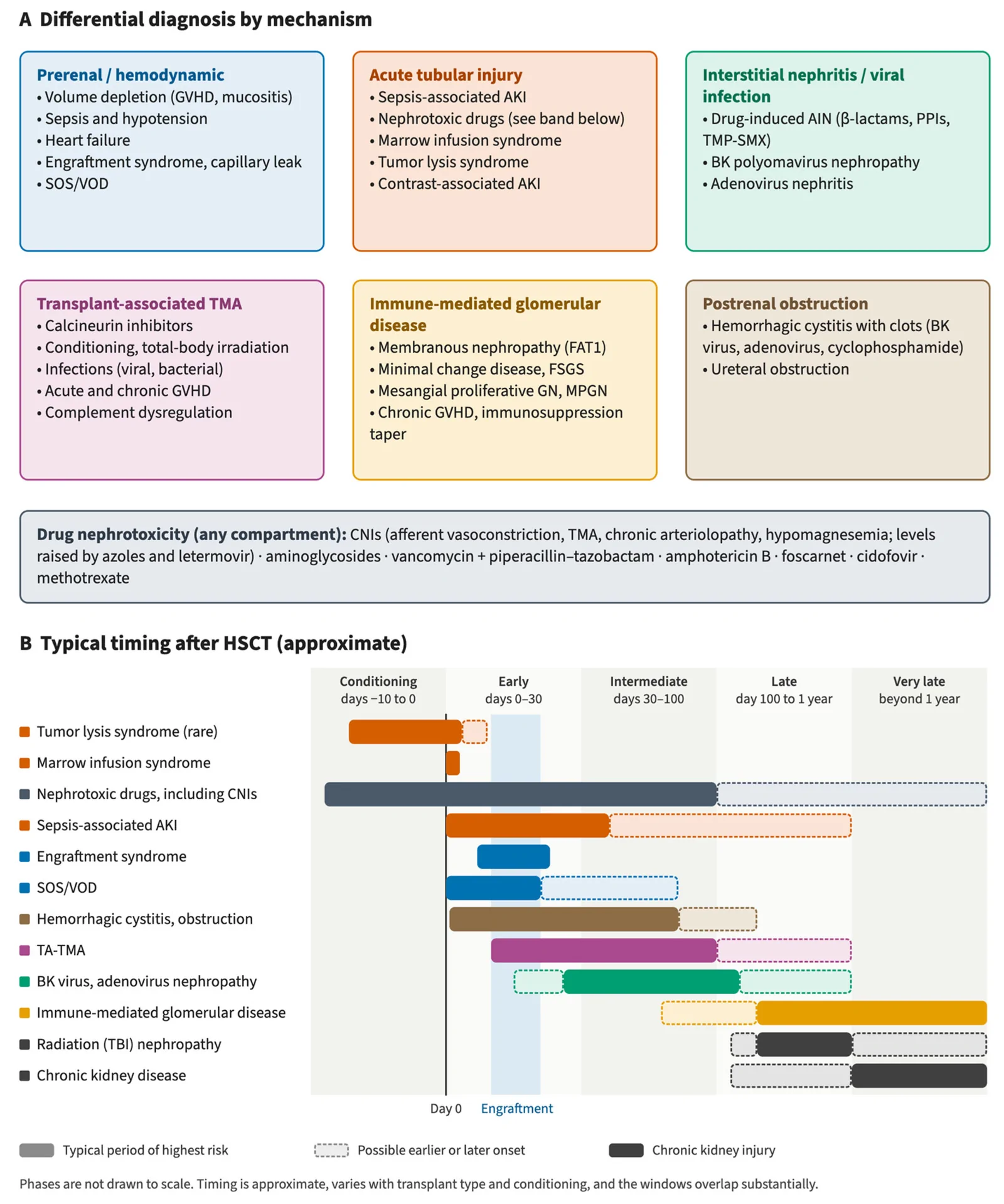
Diagnostic framework and temporal pattern of kidney complications after hematopoietic stem cell transplantation. (**A**) Differential diagnosis by mechanism; drug nephrotoxicity can affect any compartment. (**B**) Approximate timing of each complication; solid bars show the typical period of highest risk, and dashed bars indicate possible earlier or later onset. AIN, acute interstitial nephritis; AKI, acute kidney injury; CNI, calcineurin inhibitor; FSGS, focal segmental glomerulosclerosis; GN, glomerulonephritis; GVHD, graft-versus-host disease; HSCT, hematopoietic stem cell transplantation; MPGN, membranoproliferative glomerulonephritis; PPI, proton pump inhibitor; SOS/VOD, sinusoidal obstruction syndrome/veno-occlusive disease; TA-TMA, transplant-associated thrombotic microangiopathy; TBI, total body irradiation; TMA, thrombotic microangiopathy; TMP-SMX, trimethoprim–sulfamethoxazole.

Figure 1 summarizes the main causes of renal dysfunction following stem cell transplantation along with the timelines.

### 3.1. Sepsis

HSCT recipients are immunocompromised and may develop infections that lead to sepsis complicated by AKI [7,8,10]. Sepsis-associated AKI is defined as AKI by KDIGO criteria occurring within 7 days of sepsis diagnosis, and it is driven by several mechanisms, including systemic and renal inflammation, complement activation, dysregulation of the renin–angiotensin–aldosterone system, mitochondrial dysfunction, and macro- and microcirculatory dysfunction [33]. Furthermore, antimicrobial medications used in the management of sepsis, such as aminoglycosides and vancomycin, can be nephrotoxic and warrant consideration of TDM [34].

### 3.2. Nephrotoxic Medications

Discontinuation of nephrotoxic agents is the primary intervention. As mentioned above, sepsis is one of the complications of HSCT, and some of the antibiotics used to treat it can be nephrotoxic [35]. Amphotericin B, an antifungal agent, can cause vasoconstriction of the renal vasculature, resulting in hypoperfusion and renal tubular epithelial damage; the risk can be mitigated by restricting amphotericin use to documented fungal infections and by using antifungals with minimal nephrotoxicity, such as itraconazole, fluconazole and voriconazole. Acyclovir, an antiviral used for the prophylaxis and treatment of herpes simplex and varicella-zoster virus infections, can cause crystal formation in the renal tubules and collecting ducts, resulting in obstruction, especially with high-dose intravenous administration; slower intravenous administration, hydration and renal dose adjustment are recommended to reduce this risk [35]. CNIs are important medications after HSCT, but they may cause renal vasoconstriction secondary to renin–angiotensin system activation, increased production of vascular endothelial growth factor (VEGF), downregulation of renal Klotho with increased oxidative stress causing renal endothelial damage, thrombotic microangiopathy, and impaired glucose metabolism. In patients with CKD due to CNI exposure, the drug should be discontinued if possible, and switching to an alternative immunosuppressant, such as sirolimus, has been suggested [35,36].

### 3.3. Marrow Infusion Syndrome

Marrow infusion syndrome is an early complication of cryopreserved grafts, which are usually autologous, and occurs within 24–48 h after transplantation. During infusion, the patient receives the cryoprotectant dimethyl sulfoxide (DMSO), considered the main cause of infusion-related reactions [37], together with red blood cells lysed during freezing and thawing. Clinical manifestations include fever, nausea and vomiting, and hemoglobin pigment nephropathy [14]. AKI develops through multiple mechanisms, including renal vasoconstriction, direct tubular cytotoxicity from free hemoglobin, and intratubular hemoglobin cast formation [14]. Hemolysis after a major ABO-incompatible allogeneic graft is a separate entity: the recipient’s anti-A or anti-B antibodies lyse donor red cells contained in the graft, which is why such grafts are red cell-depleted before infusion [38].

### 3.4. Capillary Leak Syndrome

Capillary leak syndrome (CLS) is an early complication of HSCT, characterized by cytokine-mediated endothelial injury and increased vascular permeability. It typically occurs within 15 days of transplantation and may present with weight gain, ascites, pleural effusions, and hemodynamic instability. AKI results primarily from intravascular volume depletion and systemic inflammation [36].

### 3.5. Hepatic Sinusoidal Obstruction Syndrome

Hepatic sinusoidal obstruction syndrome (SOS; formerly known as VOD) after stem cell transplantation is an important cause of AKI, typically developing within the first 21 days after HSCT (later onset is possible), particularly following allogeneic transplantation. It results from toxic injury to hepatic sinusoidal endothelial cells caused by conditioning chemotherapy and/or radiation, leading to endothelial activation, loss of barrier integrity, and detachment. Sloughed endothelial cells, inflammatory cell infiltration, and local thrombosis obstruct the hepatic sinusoids and terminal venules, resulting in sinusoidal congestion, portal hypertension, and centrilobular hepatic necrosis.

Risk factors for SOS can be broadly categorized into patient-related factors, including extremes of age, advanced-stage malignancy, and the underlying disease, as well as baseline hepatic dysfunction, reflected by elevated transaminases, ferritin, or bilirubin levels [39]. Cytoreductive regimens including high-dose TBI, busulfan, and cyclophosphamide are presumed triggers [40].

The AKI that accompanies SOS/VOD resembles hepatorenal syndrome. SOS/VOD typically manifests within 21 days of transplantation, but late onset can occur [14]. It is characterized by edema, jaundice, hepatomegaly, and diuretic-resistant ascites. Updated diagnostic criteria were published by the European Society for Blood and Marrow Transplantation in 2023, aiming for early detection and severity grading (Table 2) [41].

The mainstay of SOS/VOD treatment has been symptom management and supportive care. Although these measures are still important, the emergence of defibrotide as an approved therapy for SOS has been associated with improved survival: in a phase III study with historical controls, day +100 survival was 38.2% vs. 25.0% [43]. Defibrotide is approved in the United States for adults and children with SOS/VOD and renal or pulmonary dysfunction after HSCT, and in the European Union for severe SOS/VOD after HSCT from 1 month of age [44,45]. Defibrotide is an endothelial-protective agent with anti-inflammatory, antithrombotic, and profibrinolytic properties [46]. It is administered at 25 mg/kg/day in four divided doses and does not require dose adjustment in patients with renal impairment or those receiving dialysis [47], and early initiation after diagnosis has been associated with improved day +100 survival [48]. Although earlier pediatric and retrospective studies suggested a potential role for prophylaxis in high-risk patients, the phase III HARMONY trial did not demonstrate improvement in SOS/VOD-free survival at day 30 after HSCT with defibrotide prophylaxis compared with best supportive care (67% vs. 73%, respectively) [49].

### 3.6. GVHD-Associated Kidney Disease

Allogeneic HSCT, including bone marrow transplantation (BMT), may cause GVHD. However, involvement of the kidney in GVHD is not well understood, and there are no established histopathologic criteria for GVHD-associated kidney disease. Experimental and clinical studies support the kidney as a target of GVHD. Immune-mediated endothelial injury, T-cell infiltration, tubulitis, peritubular capillaritis, glomerulitis, and proinflammatory cytokine activation have been suggested to contribute to renal damage [50,51,52]. These lesions are not specific: kidney injury after HSCT is often multifactorial, biopsy findings overlap with those of drug toxicity, ischemia, infection, thrombotic microangiopathy and radiation injury, and a kidney biopsy is needed to establish the cause [50,51]. Overall, GVHD-associated kidney disease is considered rare compared with GVHD involving other organs, with reported estimates of approximately 1% among allogeneic HSCT recipients [53].

### 3.7. Glomerular Disease After HSCT

Glomerular disease after HSCT overlaps with, but is not limited to, GVHD-associated kidney disease: in the cohort of Yap et al., 31 of 2204 recipients (1.4%) developed de novo glomerular disease, and 48.4% had pre-existing or concurrent GVHD at renal presentation [54]. It can be grouped by clinical setting: disease associated with chronic GVHD, disease appearing during immunosuppression taper, de novo immune-mediated disease, and thrombotic microangiopathy lesions that mimic glomerulonephritis (Section 3.8). In that cohort, the biopsy spectrum included thrombotic microangiopathy (38.7%), membranous nephropathy (25.8%), mesangial proliferative glomerulonephritis (12.9%), minimal change disease (9.7%), focal segmental glomerulosclerosis (9.7%), and membranoproliferative glomerulonephritis (3.2%) [54]. Among patients who developed nephrotic syndrome after allogeneic HSCT, membranous nephropathy is the most common histopathological lesion, accounting for approximately two-thirds of reported cases [55]. It typically presents late after transplantation, with a median onset of 20 months. It is frequently associated with chronic GVHD or tapering of immunosuppression. The optimal treatment strategy for this rare manifestation remains undefined [56]; however, successful use of rituximab and CNIs has been reported in case reports and series [52]. More recently, protocadherin FAT1 has emerged as a potential target antigen in membranous nephropathy after HSCT [57]. The reported response to rituximab in FAT1-positive cases suggests that identifying the underlying antigen may help guide treatment and further supports an immune-mediated mechanism [58].

### 3.8. Transplant-Associated Thrombotic Microangiopathy

Transplant-associated thrombotic microangiopathy (TA-TMA) is a serious complication of HSCT. Endothelial injury is the central pathogenic event, triggering complement activation, microvascular thrombosis, microangiopathic hemolytic anemia, thrombocytopenia, and progressive multiorgan dysfunction, with the kidneys, lungs, gastrointestinal tract, and central nervous system being particularly affected. Renal involvement is often an early manifestation and may present with new or worsening hypertension, proteinuria, and AKI, frequently preceding the development of overt hematologic abnormalities [59]. Incidence varies considerably because of differences in the diagnostic criteria applied [60]. In the prospective MIDAS consortium study of 239 adult allogeneic recipients, TA-TMA occurred in 56.9%, with severe disease affecting 21.8% by day +100. Severe TA-TMA was associated with higher non-relapse mortality (42.1% at day +180) compared with non-severe disease (8.4%) and patients without TA-TMA (5.8%) [61]. It is more frequent after allogeneic transplantation but can also develop after autologous transplantation. Risk factors include unrelated or HLA-mismatched donors, older recipient age, female sex, CNI exposure, MAC, viral infections, and GVHD [62,63]. MIDAS findings showed that TA-TMA can develop independently of acute GVHD, although its incidence increases with GVHD severity. Higher pre-transplant creatinine, day +28 suppression of tumorigenicity 2 (ST2), and the Endothelial Activation and Stress Index (EASIX) were associated with TA-TMA risk or adverse outcomes, whereas soluble C5b-9 did not significantly predict severe disease in adults [64]. According to the 2023 international harmonized consensus definition, TA-TMA is diagnosed by histopathological evidence on kidney or gastrointestinal biopsy, or by the presence of at least four of seven features documented at two consecutive time points within 14 days: anemia, thrombocytopenia, elevated lactate dehydrogenase (LDH), schistocytes, hypertension, proteinuria, and elevated soluble C5b-9. High-risk features include LDH > 2 times the upper limit of normal, urine protein-to-creatinine ratio ≥ 1 mg/mg, elevated soluble C5b-9, organ dysfunction other than KDIGO stage 1 AKI, concurrent grade II–IV acute GVHD, or concurrent bacterial or viral infection [65]. The 2023 consensus recommends screening all allogeneic recipients at least weekly until day +100 with a complete blood count, LDH, random urine protein-to-creatinine ratio and a blood smear, and continuing beyond day +100 while risk factors persist [65]. In MIDAS, a rise in creatinine by day +7 and hypertension by day +14 were early signs of severe TA-TMA [61]. The various published diagnostic criteria are outlined in Table 3. 

Management of TA-TMA includes both supportive and targeted therapy (Table 4). Supportive management includes blood pressure control, correction of volume and electrolyte abnormalities, avoidance of additional nephrotoxic exposure and early identification and treatment of viral infections. Modification or discontinuation of CNIs should be individualized, as reducing immunosuppression may increase the risk of GVHD. Plasma exchange was historically used in the management of TA-TMA; however, clinical responses have been inconsistent, and accumulating evidence suggests that the risks and procedural burden may outweigh the potential benefits [60].

In children and young adults, eculizumab has been associated with higher survival than historical controls [70]; however, a retrospective adult study reported no improvement in clinical response (56% vs. 71% with conventional treatment) and higher non-relapse mortality, with deaths mainly attributed to infections [71]. In addition, a phase III randomized trial of ravulizumab in adults and adolescents did not meet its primary endpoint [72]. Apart from this trial, the evidence for complement inhibition comes from single-arm and retrospective studies [73]; better survival than historical controls has been reported mainly in children and young adults, the adult data are limited and conflicting, and no randomized trial has yet confirmed a benefit over best supportive care.

Narsoplimab, a monoclonal antibody targeting mannan-binding lectin-associated serine protease-2 (MASP-2), became the first therapy approved by the U.S. Food and Drug Administration (FDA) specifically for TA-TMA in December 2025, for patients aged ≥2 years [74]. In the European Union, the Committee for Medicinal Products for Human Use issued a negative opinion in June 2026 and confirmed it on re-examination in October 2026, so narsoplimab is not authorized there [75]. The pivotal single-arm study of narsoplimab demonstrated a 61% response rate, with improvement in organ function in 74% of patients [76]. Expanded-access studies have reported further response and survival data in children and adults [77,78]. In patients aged ≥16 years with high-risk TA-TMA, mortality with narsoplimab was lower than in an external control group from the Kyoto Stem Cell Transplantation Group registry (HR 0.28, 95% CI 0.22–0.37, for trial and expanded-access patients combined) [79]. However, these data come from a single-arm trial of 28 adults, expanded-access programs and a comparison with external controls, so randomized studies are still needed, particularly in adults. Overall, complement-directed therapies have expanded the treatment options for TA-TMA, and the choice of agent, patient selection and timing remain to be defined in prospective studies [73].

**Table 4:** Complement-directed therapies for transplant-associated thrombotic microangiopathy (TA-TMA), grouped by level of evidence.

Therapy (Target)	Population and Design	Main Findings	Status
Randomized controlled trial			
Ravulizumab (C5)	Adults and adolescents aged ≥12 years; phase III, double-blind, placebo-controlled (NCT04543591; *n* = 146) [72]	Primary endpoint (event-free survival through 26 weeks) not met [72]	Not approved for TA-TMA
Prospective single-arm trials			
Narsoplimab (MASP-2)	Adults; open-label pivotal trial (NCT02222545; *n* = 28) [76]	Response 61%; 100-day survival after diagnosis 68% [76]	FDA-approved (December 2025) for adults and children aged ≥2 years [74]; EU: not authorized (negative CHMP opinion June 2026, confirmed on re-examination October 2026) [75]
Eculizumab (C5)	Children and young adults with high-risk TA-TMA; multicenter study (NCT03518203; 21 evaluable patients) [70]	Survival 71% at 6 months after diagnosis and 62% at 1 year after HSCT, vs. 18% and 16.7% in historical controls [70]	Not approved for TA-TMA (off-label use)
Ravulizumab (C5)	Children; phase III trial (NCT04557735; *n* = 41) [80]	Complete TMA response 17.1%; survival 87.2% at 26 weeks and 73.4% at 52 weeks [80]	Not approved for TA-TMA
Retrospective cohorts and expanded access			
Narsoplimab (MASP-2)	Children and adults; global expanded-access program (*n* = 136) [77]	Allogeneic recipients with high-risk TA-TMA treated first line: 1-year survival 75.0% in children (*n* = 12) and 58.0% in adults (*n* = 49) [77]	As above
Eculizumab (C5)	Children with high-risk TA-TMA; cohorts of 64 [81] and 29 [82] patients	1-year survival after HSCT 66% vs. 16.7% in an untreated historical cohort [81]; response 65.5% and 1-year survival 55.2% [82]	Not approved for TA-TMA (off-label use)
Eculizumab (C5)	Adults with high-risk TA-TMA; comparison with conventional therapy (16 vs. 31 patients) [71]	Response 56% vs. 71%; higher non-relapse mortality with eculizumab, with deaths mainly from infections [71]	Not approved for TA-TMA (off-label use)
Investigational agents without published efficacy data			
Pegcetacoplan (C3)	Adults; phase II trial (NCT05148299; *n* = 12), completed	Results not yet published in a peer-reviewed journal	Investigational
Iptacopan (factor B)	Age ≥12 years, second line; phase II trial (NCT07347990)	Recruiting (since August 2026)	Investigational

Abbreviations: C3, complement component 3; C5, complement component 5; CHMP, Committee for Medicinal Products for Human Use; EU, European Union; FDA, U.S. Food and Drug Administration; HSCT, hematopoietic stem cell transplantation; MASP-2, mannan-binding lectin-associated serine protease-2; NCT, ClinicalTrials.gov identifier; TMA, thrombotic microangiopathy. Response definitions differ between studies. Regulatory and trial status as of October 2026.

### 3.9. Other Causes of AKI

#### 3.9.1. Virus-Associated Kidney Injury

BK polyomavirus and adenovirus are important causes of urinary tract complications following HSCT, particularly in allogeneic recipients. BK virus commonly causes hemorrhagic cystitis, which may lead to AKI through urinary obstruction by blood clots. Less commonly, BK virus can cause tubulointerstitial nephritis [83]. Similarly, adenovirus may cause hemorrhagic cystitis or, rarely, necrotizing tubulointerstitial nephritis [84]. Diagnosis relies on clinical assessment, viral testing, imaging when obstruction is suspected, and occasionally kidney biopsy [83]. Both BK virus and adenovirus have been associated with development of TA-TMA [36]. Management is primarily supportive, as antiviral treatment options remain limited by uncertain efficacy and potential nephrotoxicity [83,85].

#### 3.9.2. Postrenal Obstruction

Although less common, postrenal obstruction is an important reversible cause of AKI following HSCT. Hemorrhagic cystitis, particularly related to BK virus reactivation, may lead to clot retention or ureteral obstruction with hydronephrosis. Early recognition through bladder assessment and renal ultrasonography is essential, as severe cases may require urological intervention [85].

## 4. Long-Term Kidney Outcomes After HSCT

With advancements in conditioning regimens, supportive care, and management of GVHD, long-term survivorship after HSCT has improved substantially. The long-term consequences of AKI have emerged as a major determinant of late morbidity, cardiovascular risk, and mortality [86]. Renal outcomes are no longer a temporary concern confined to the acute post-transplant hospitalization but a core component of survivorship care, warranting dedicated, longitudinal attention from the onconephrologist working alongside transplant hematologists.

### 4.1. Transition to Chronic Kidney Disease

AKI following HSCT is not always a self-limited event confined to the peri-transplant period. In the general AKI literature, an AKI episode is viewed as an inflection point toward progressive nephron loss; this model rests mainly on non-HSCT data. Incomplete tubular repair, peritubular capillary rarefaction, maladaptive fibrosis, pericyte-to-myofibroblast transition, and persistent low-grade inflammation following an AKI episode create a substrate for irreversible nephron loss even when serum creatinine appears to normalize [87,88,89,90]. In HSCT cohorts, AKI is associated with later CKD: among 408 adult allogeneic recipients, 19% had CKD (eGFR < 60 mL/min/1.73 m^2^) at 1 year, and AKI in the first 100 days was associated with CKD (adjusted odds ratio 3.86, 95% CI 1.70–8.73) [91]. 

Historically, a late-onset renal syndrome termed chronic BMT nephropathy was described after HSCT [92,93,94,95]. This entity typically manifests 6–12 months after transplantation and is characterized by hypertension, anemia disproportionate to the degree of renal impairment, and azotemia, often accompanied by proteinuria and microangiopathic changes on kidney biopsy resembling radiation nephritis [93,94,96]. TBI is the dominant risk factor. Reduction and hyperfractionation of the total dose and partial renal shielding have been associated with a lower incidence [97]. 

The cumulative incidence of CKD varies from 7% to 48%, and it may develop between 6 months and 10 years post-HSCT, with ∼4% of cases progressing to kidney failure [5]. Its pathogenesis is multifactorial and includes antecedent AKI, TA-TMA, CNI nephrotoxicity, chronic GVHD-associated glomerular disease (including membranous nephropathy and minimal change disease), and radiation-related renal injury. Importantly, AKI itself is an established risk factor for subsequent CKD, suggesting that early renal insults are an important contributor to the later development of persistent kidney dysfunction [5]. The presence of subsequent CKD may limit therapeutic options for both chronic GVHD management and subsequent disease relapse, and as such, has been associated with worse GVHD-free/relapse-free survival [91].

### 4.2. Surveillance and Long-Term Nephrology Follow-Up

Given the latency and insidious onset of post-HSCT renal disease, regular and structured long-term surveillance is essential. This should ideally begin before conditioning with a baseline assessment of renal function, blood pressure, and urinalysis to serve as a reference point for subsequent monitoring. The 2023 international survivorship recommendations, developed by expert-panel consensus, advise checking serum creatinine and blood urea nitrogen, urinalysis and the urine albumin-to-creatinine ratio at 6 months and 1 year after transplantation and at least annually thereafter (more often after HSCT for multiple myeloma or amyloidosis), and measuring blood pressure at every survivorship visit [98]. Based on the risk factors discussed in Section 2 and Section 4.1 and on general risk factors for CKD [99], we suggest closer surveillance in patients with prior AKI, chronic GVHD, ongoing CNI exposure, TBI-based conditioning, diabetes or hypertension; of these, the link between AKI and later CKD has been reported in an adult HSCT cohort [91].

In line with the 2023 harmonized TA-TMA consensus, the screening panel (Section 3.8) should be repeated after day +100 whenever risk factors appear or persist, such as GVHD, viral infection or CNI exposure [65], and blood pressure should be measured at every visit [98]. Early recognition allows prompt management of triggers and, in high-risk disease, consideration of targeted therapy (Table 4). Long-term follow-up should also proactively address the downstream metabolic consequences of CKD (hypertension, anemia, mineral and bone disease, and metabolic acidosis). Nephrology referral thresholds have not been defined for HSCT survivors. The KDIGO 2024 criteria for the general CKD population (eGFR < 30 mL/min/1.73 m^2^, a sustained fall in eGFR of more than 20%, albuminuria > 700 mg/g or ≥300 mg/g with hematuria, a doubling of albuminuria, or hypertension uncontrolled on ≥4 drugs) [99] may identify these patients late, because CKD with an eGFR < 60 mL/min/1.73 m^2^ at 1 year after allogeneic HSCT is already associated with lower overall survival [91].

## 5. Conclusions

AKI and CKD are frequent complications after HSCT, arise from multiple and often overlapping etiologies, and are associated with adverse short- and long-term outcomes. Early recognition of kidney injury and transplant-specific complications, together with appropriate long-term surveillance, is essential to reduce progressive kidney dysfunction. Multidisciplinary collaboration between transplant hematologists and nephrologists is critical to improving kidney and overall outcomes in this patient population.

## Figures and Tables

**Table 1:** Transplant- and patient-related risk factors for AKI after HSCT.

**Transplant-Related Risk Factors**	
Before HSCT	Allogeneic transplantation
	Myeloablative conditioning *
	Specific conditioning regimens, including fludarabine–melphalan
	Total body irradiation *
	HLA mismatch/donor characteristics
After HSCT	GVHD prophylaxis strategy, including CNI exposure
	Concomitant nephrotoxic medications (e.g., aminoglycosides, NSAIDs, selected antivirals/antifungals)
	Acute GVHD
	Infectious complications/sepsis
	CMV reactivation
	SOS/VOD
	TA-TMA
**Patient-Related Risk Factors**	
Before HSCT	Older age
	Pre-existing CKD/reduced baseline kidney function
	Underlying disease
	Comorbidity (HCT-CI)
	Low serum albumin

* Associations between conditioning intensity, TBI, and AKI are heterogeneous across studies and are influenced by patient selection, transplant characteristics, GVHD prophylaxis, and post-transplant complications. Abbreviations: AKI, acute kidney injury; CKD, chronic kidney disease; CMV, cytomegalovirus; CNI, calcineurin inhibitor; GVHD, graft-versus-host disease; HCT-CI, Hematopoietic Cell Transplantation-Comorbidity Index; HLA, human leukocyte antigen; HSCT, hematopoietic stem cell transplantation; NSAIDs, nonsteroidal anti-inflammatory drugs; SOS/VOD, sinusoidal obstruction syndrome/veno-occlusive disease; TA-TMA, transplant-associated thrombotic microangiopathy; TBI, total body irradiation.

**Table 2:** Refined EBMT criteria for sinusoidal obstruction syndrome/veno-occlusive disease (SOS/VOD) in adults: (**a**) diagnosis; (**b**) severity grading, applied once the diagnosis is made. Adapted from Mohty et al. [41].

**(a) Diagnosis**				
**Category**	**Criteria**			
Time of onset	Classical: within 21 days of HSCT (original Baltimore criteria). Late onset: beyond day 21; hyperbilirubinemia is not mandatory for diagnosis.			
Probable	≥2 of: bilirubin ≥ 2 mg/dL, painful hepatomegaly, weight gain >5%, ascites, or ultrasound and/or elastography suggestive of SOS/VOD			
Clinical	Bilirubin ≥ 2 mg/dL plus ≥2 of: painful hepatomegaly, weight gain >5%, ascites			
Proven	Histology, or hepatic venous pressure gradient (HVPG) ≥ 10 mmHg			
Exclusion	In all patients, these signs and symptoms should not be attributable to other causes.			
**(b) Severity Grading**				
**Parameter**	**Mild ^a^**	**Moderate ^a^**	**Severe**	**Very Severe (MOD) ^b^**
Time since first clinical symptoms	>7 days	5–7 days	≤4 days	Any time
Bilirubin, mg/dL (µmol/L)	≥2 and <3 (≥34 and <51)	≥3 and <5 (≥51 and <85)	≥5 and <8 (≥85 and <136)	≥8 (≥136)
Bilirubin kinetics	–	–	Doubling within 48 h	–
Transaminases	≤2× normal	>2 and ≤5× normal	>5 and ≤8× normal	>8× normal
Weight increase	–	–	≥5%	≥10%
Creatinine vs. baseline at transplant	At baseline	<1.5×	≥1.5 and <2×	≥2×, or other signs of MOD

A patient takes the grade in which ≥2 criteria are met; if criteria fall in different grades, the more severe grade applies. ^a^ With ≥2 risk factors for SOS/VOD, the patient moves up one grade. ^b^ Any patient with MOD is very severe. MOD is dysfunction of ≥2 organs on the Sequential Organ Failure Assessment (SOFA) score (score ≥2, or a rise of ≥2). Because bilirubin is usually ≥2 mg/dL, one organ besides the liver with dysfunction ≥ 2 is enough. Platelet count only if their score rises by ≥2. Children are assessed with separate EBMT pediatric criteria [42]. Abbreviations: EBMT, European Society for Blood and Marrow Transplantation; HSCT, hematopoietic stem cell transplantation; HVPG, hepatic venous pressure gradient; MOD, multiorgan dysfunction; SOFA, Sequential Organ Failure Assessment.

**Table 3:** Diagnostic criteria for transplant-associated thrombotic microangiopathy (TA-TMA): the 2023 harmonized definition compared with earlier criteria.

Diagnostic Feature	2023 Harmonized Definition (EBMT, ASTCT, APBMT, CIBMTR) [65] ^a^	International Working Group (IWG) Criteria (LeukemiaNet) [66]	Blood and Marrow Transplant Clinical Trials Network (BMT CTN) Criteria [67]	Cho et al. Probable TA-TMA Criteria [68]	Jodele et al. Criteria [69]
Diagnosis requires	Biopsy (kidney or GI), or ≥4 of 7 features (anemia, thrombocytopenia, LDH, schistocytes, hypertension, proteinuria, sC5b-9) within 14 days at two consecutive time points	All 5 criteria present simultaneously	All 4 criteria present simultaneously	All criteria present at ≥2 time points	Renal biopsy, or ≥4 of 7 features at ≥2 time points within 14 days
Basis and population	Expert consensus (modified Jodele criteria); adults and children, with age-specific blood pressure thresholds	Expert consensus	Expert consensus	Retrospective cohort of 672 allogeneic transplant recipients	Prospective cohort of 100 consecutive transplant recipients; children and young adults
LDH	>ULN for age	Increased	Increased	Increased	Increased
Schistocytes	Present	>4%	≥2/HPF	≥2/HPF	Microangiopathy (schistocytes or tissue biopsy)
Thrombocytopenia	Failed platelet engraftment, higher-than-expected transfusion needs, refractoriness to platelet transfusion, or ≥50% fall after full engraftment	Present	Not required	Present	Present
Hemoglobin	Failure to achieve transfusion independence despite neutrophil engraftment, Hb fall ≥1 g/dL, or new transfusion dependence; other causes (e.g., autoimmune hemolytic anemia, pure red cell aplasia) excluded as the sole cause	Decreased or increased RBC transfusion requirement	Not required	Decreased	Decreased or increased RBC transfusion requirement
Coombs test	Not required ^b^	Not required	Negative (direct and indirect)	Negative	Not required
Haptoglobin	Not required	Decreased	Not required	Decreased	Not required
Coagulation profile	Not required	Not required	Not required	Normal	Not required
Renal or neurologic involvement	Proteinuria (rUPCR ≥ 1 mg/mg) and hypertension (≥140/90 mmHg if ≥18 y; >99th percentile if <18 y), each a separate feature	Not required	Required: renal and/or neurologic dysfunction without other explanation	Not required	Proteinuria ≥ 30 mg/dL and hypertension, each a separate feature
Terminal complement activation	Soluble C5b-9 > ULN	Not required	Not required	Not required	Elevated soluble C5b-9

Abbreviations: APBMT, Asia-Pacific Blood and Marrow Transplantation Group; ASTCT, American Society for Transplantation and Cellular Therapy; BMT CTN, Blood and Marrow Transplant Clinical Trials Network; CIBMTR, Center for International Blood and Marrow Transplant Research; EBMT, European Society for Blood and Marrow Transplantation; GI, gastrointestinal; GVHD, graft-versus-host disease; Hb, hemoglobin; HPF, high-power field; IWG, International Working Group; KDIGO, Kidney Disease: Improving Global Outcomes; LDH, lactate dehydrogenase; RBC, red blood cell; rUPCR, random urine protein-to-creatinine ratio; sC5b-9, soluble C5b-9; ULN, upper limit of normal. ^a^ High-risk TA-TMA under the 2023 definition: any of elevated sC5b-9 (>ULN), peak LDH > 2 × ULN, rUPCR ≥ 1 mg/mg, organ dysfunction other than KDIGO stage 1 acute kidney injury, concurrent grade II–IV acute GVHD, or concurrent bacterial or viral infection. ^b^ A positive Coombs test (e.g., after intravenous immunoglobulin or with ABO incompatibility) does not by itself exclude TA-TMA.

## Data Availability

No new data were created or analyzed in this study. Data sharing is not applicable to this article.
